# Multifunctional Nanotechnology-Enabled Sensors for Rapid Capture and Detection of Pathogens

**DOI:** 10.3390/s17092121

**Published:** 2017-09-15

**Authors:** Fatima Mustafa, Rabeay Y. A. Hassan, Silvana Andreescu

**Affiliations:** 1 Department of Chemistry and Biomolecular Science, Clarkson University, Potsdam, NY 13699-5810, USA; mustaffm@clarkson.edu; 2 Applied Organic Chemistry Department, National Research Centre (NRC), El Bohouth st., Dokki, 12622-Giza, Egypt; rabeayy@yahoo.com

**Keywords:** multifunctional nanotechnology, pathogens, portable sensors, integrated sensing systems, toxicity, food

## Abstract

Nanomaterial-based sensing approaches that incorporate different types of nanoparticles (NPs) and nanostructures in conjunction with natural or synthetic receptors as molecular recognition elements provide opportunities for the design of sensitive and selective assays for rapid detection of contaminants. This review summarizes recent advancements over the past ten years in the development of nanotechnology-enabled sensors and systems for capture and detection of pathogens. The most common types of nanostructures and NPs, their modification with receptor molecules and integration to produce viable sensing systems with biorecognition, amplification and signal readout are discussed. Examples of all-in-one systems that combine multifunctional properties for capture, separation, inactivation and detection are also provided. Current trends in the development of low-cost instrumentation for rapid assessment of food contamination are discussed as well as challenges for practical implementation and directions for future research.

## 1. Introduction

Continued interest in human health and food safety has driven the innovation in the development of technology for the rapid assessment of toxicity risks due to presence of harmful molecules and toxins affecting the quality of food products and the environment. The Centers for Disease Control and Prevention (CDC) in the USA estimates that approximately 48 million people are affected by diseases caused by bacteria, viruses and parasites [1]. Statistics worldwide indicate 600 million foodborne illnesses with 420,000 deaths in 2010, according to a report issued by WHO Foodborne Disease Burden Epidemiology Reference Group (FERG). Pathogenic bacteria are some of the most threatening organisms [2], with *Salmonella* spp., *Campylobacter* spp. and *Escherichia coli* (*E. coli*) as the primary pathogens responsible for most outbreaks in the US [3,4,5,6]. The large number of reported foodborne outbreaks and the economic and social implications require analytical methodologies that can provide rapid screening and identification of pathogen and toxins in a timely manner. Conventional analytical methods are often cumbersome and cannot be used directly in the field. Methods for detection of bacterial pathogens such as polymerase chain reaction (PCR) and plate counting are time and labor extensive and they usually require further enrichment and amplification [7]. Rapid and simple methods that can be used in the field with minimum reagents and power requirement are more desirable for rapid field screening and quantification of samples [8,9]. The basic properties of the different types of nanostructures enable use of these materials for pathogen detection and integration with biomolecules [10,11]. Besides their intensive use in the development of bioassays and sensors, several types of nanostructures fabricated from materials such as silver, copper, gold/silver-tellurium in various configurations including nanowires, nanotubes, nano- particles (NPs) and nanoarrays have been reported for their antimicrobial activity [12,13,14,15,16,17]. Thus, the development of sensors and smart labels as indicators of toxicity and multifunctional systems that combine capture, detection and inactivation functions has recently become an important area of research [18,19,20]. Such properties can be achieved by integrating nanosized materials with unique multifunctional properties [21]. The high surface-area-to-volume ratio and the nanosize properties can be tailored to change in response to a target making them attractive for designing multifunctional sensing systems [22]. For example, NPs such as silver (AgNPs) can be used to prevent bacterial infections, and in the same time enable detection and inactivation of bacteria [18]. These can be added to surfaces and coatings to create dual antimicrobial and sensing systems. To achieve selectivity, nanomaterials are conjugated with biological and molecular receptors that have the ability to bind and enrich the target and improve detection sensitivity. Commonly used are antibodies [23,24,25,26,27,28], enzymes [29], DNA [30,31,32,33], phages [34,35,36,37,38,39,40,41,42,43], biologically derived materials such as aptamers [19,44,45,46,47,48,49,50,51,52,53,54,55], synthetic antimicrobial polypeptides [56], recombinant antibodies [57] and biomimetic molecules like molecular imprinted polymers (MIPs) [58,59,60]. Nanomaterials have been used in sensing platforms to enhance sensor performance by providing the actual signal [25,27,50,51], for signal amplification [23,46,52,53,54,61,62] and labeling purposes [19,45,48,54] as well as for concentration and separation [26,34,48]. While a regulatory framework is being developed [63], there is an increased trend to implement nanomaterials in food applications, smart packaging and consumer products [64,65,66,67]. 

The aim of this review is to provide a critical overview of the different types of bio-functionalized nanomaterials that can be used to develop smart multifunctional sensors and labels for rapid capture, detection and screening of pathogens and toxins. We discuss their assembly in portable sensing platforms and provide examples of real world applications. Opportunities of these technologies as well as possible risks and challenges for implementation are discussed. 

## 2. Nanotechnology for Detection of Pathogens and Toxins: Opportunities and Challenges

The combination of nanotechnology with biosciences, electronics and software engineering has enabled the development of novel systems that are capable of providing selective and specific information on the presence and amount of pathogens and toxins [22]. Nanomaterials-based sensing approaches provide opportunities for miniaturization, increased portability, large scale production and cost reduction for rapid measurement and screening applications [68]. A variety of systems have been designed to measure specific target analyses, indicate a total toxicity or provide a general product quality status during transport or storage, as well as a nutritional content. Others can be used for product authentication or detection of food adulteration. By carefully selecting sensing materials, additional capabilities can be achieved to increase shelf-life [66,67]. Examples of nano-based biotechnological approaches include:

- miniaturized portable instrumentation for field testing
- smart labels to indicate quality and safety
- smart packaging and coatings with antimicrobial and antioxidant properties to inhibit bacterial growth, enhance product safety and shelf-life [69,70]
- delivery systems of active ingredients
- nano-barcodes or trackers for product traceability and authentication

Ongoing research is dedicated to develop affordable portable systems to move away from centralized laboratory and enable faster, high throughput and lower cost analysis. Several portable sensors currently exist that measure temperature and humidity [71,72,73] in the packaging of goods for product traceability. Advanced capabilities can be achieved by integrating materials with biologically selective receptors to achieve selectivity and expand detection capabilities to analysis of specific targets associated with toxicity or freshness status [74]. Nanomaterials of various compositions ranging from metal NPs to quantum dots and carbon-based nanostructures have been interfaced with virtually all types of biomolecule, e.g., antibodies, aptamers and enzymes [68]. Artificial receptors such as synthetic peptides and molecularly imprinted polymers have also been used. Optical and electrochemical sensors are the most widely used detection modalities due to their simple operation and portability, although sensors based on micro-cantilever detection, radiofrequency identification (RFID) and quartz crystal microbalance (QCM) are also reported [75]. Despite significant progress, the development of biosensing systems for food packaging applications is still in infancy. The following sections provide an overview of the current status of bio-functionalized nanostructured interfaces and representative sensing schemes for detection of pathogens and their applications.

## 3. Nanotechnology-Enabled Sensors and Sensing Systems for Detection of Pathogens

### 3.1. Aptamer-Based Nanosensing 

Aptamers are short sequences of oligonucleotides or peptides synthesized by systematic evolution of ligands by exponential enrichment (SELEX) [76,77,78]. Due to their high specificity and affinity to a variety of targets aptamers have found many applications as bioreceptors in the development of bioanalytical assays and biosensors as a replacement for antibodies [53,76,79]. Table 1 provides a summary of the various sensing strategies involving aptamer recognition on nanomaterial supports. 

**Figure 1:**
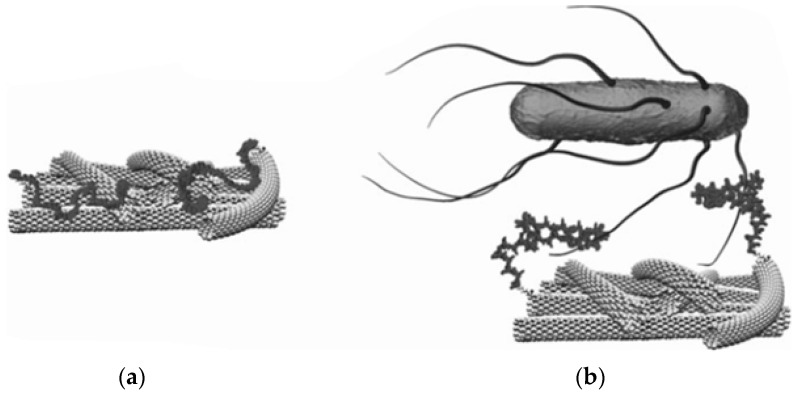
(**a**) Possible conformations of the aptamers that are self-assembled on carbon nanotubes; (**b**) Schematic representation of the interaction between the target bacteria and the hybrid aptamer–SWCNT system (adapted with permission from [53]).

**Figure 2:**
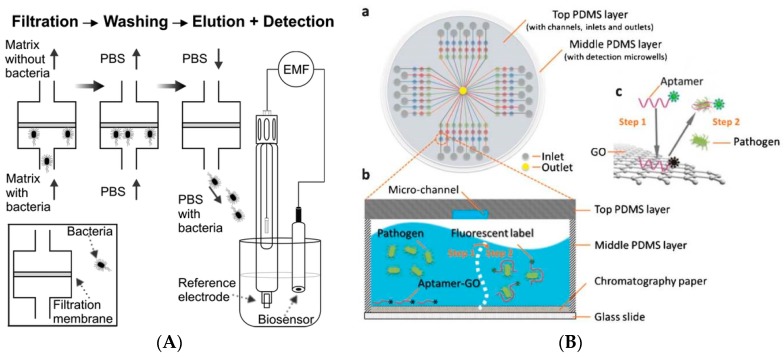
(**A**) Biosensing measurements using a potentiometric SWCNP-based aptasensor connected to a sample pretreatment system to remove the matrix in real samples and detect microorganisms. From left to right: filtration of sample and matrix removal, washing with PBS, elution with PBS and potentiometric detection of bacteria recovered in eluate (adapted with permission from [43]. Copyright (2010) American Chemical Society; (**B**) PDMS/paper hybrid microfluidic system for one-step multiplexed pathogen detection using aptamer-functionalized GO biosensors. (**a**) Microfluidic biochip layout; (**b**,**c**) illustrate the principle of the one-step ‘turn-on’ detection approach based on the interaction among GO, aptamers and pathogens. Step 1: when an aptamer is linked to the GO surface, its fluorescence is quenched. Step 2: when the target pathogen is present, the target pathogen induces the aptamer to be liberated from GO and thereby restores its fluorescence for detection (adapted from [45] with permission of The Royal Society of Chemistry).

Most materials used for designing aptasensors for pathogen detection are carbon-based (e.g., single or multi-wall carbon nanotubes (SWCNT [44], MWCNTs [52]), graphene oxide (GO)), metal NPs like Au [37], fluorescent quantum dots (QDs) [31,36] and magnetic beads [17]. Zelada-Guillén et al. [53] demonstrated potentiometric label free detection *E. coli* with a linearity response up to 10^4^ CFU/mL using SWCNT functionalized with aptamers. The assembly of aptamers on carbon nanotube hybrids was also studied using molecular dynamics (MD) simulations [44]. DNA was observed to undergo a spontaneous conformational change enabling the hybrid to self-assemble via the π–π stacking interactions [80]. In presence of bacteria, the aptamer changes its conformation from the SWCNT sidewalls, in the region that separates the phosphate groups, largely ionized at pH 7.4, inducing a change in surface charge and surface potential (Figure 1). The approach can be used for potentiometric detection of bacteria [81]. A SWCNT based potentiometric aptasensor enabled selective detection and differentiation of different strains of bacteria such as *E. coli CECT 675* as a nonpathogenic and pathogenic *E. coli O157:H* in milk and apple juice [52]. The aptasensor was connected to a pre-treatment separation system to remove the effect of matrix and control the ionic strength (Figure 2A). 

The electrical properties of carbon-based nanomaterials have been used to construct a variety of electrochemical sensors for detection of pathogens. A polydimethylsiloxane (PDMS)/paper/glass hybrid microfluidic system integrated with aptamer-functionalized GO nano-biosensors enabled rapid one-step multiplex detection of pathogens based on fluorescence quenching (Figure 2B) [54]. A detection limit of 11 CFU/mL for *Lactobacillus acidophilus* was achieved with the microchip in 10 min. The system was extended to measure simultaneously *Staphylococcus aureus* and *Salmonella enterica*.

**Figure 3:**
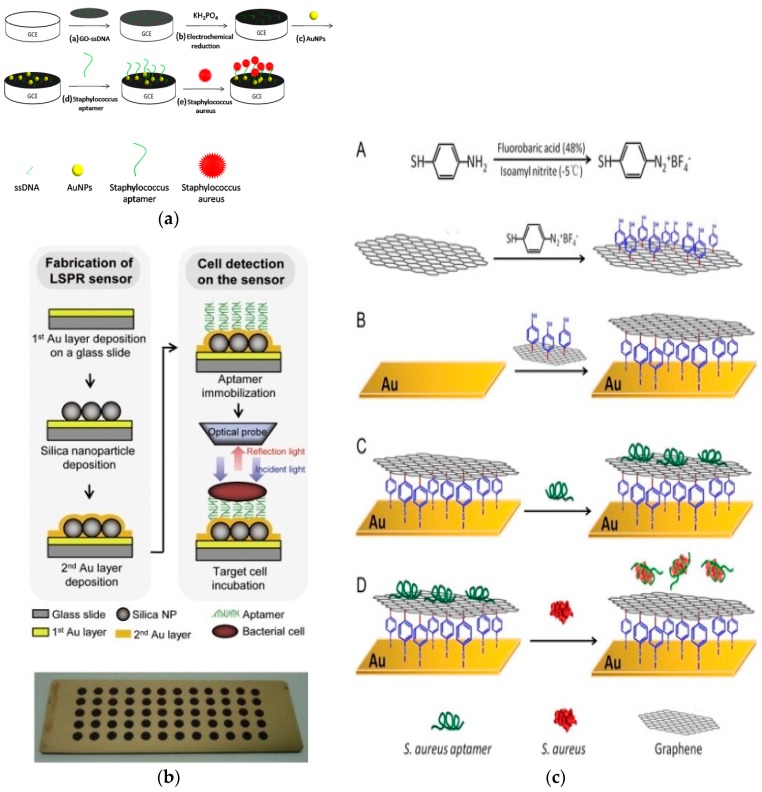
(**a**) Schematic representation of the principle of impedance-based detection of *S. aureus* on a GCE-rGO-ssDNA-AuNPs-aptamer nanocomposite (adapted with permission from [37]); (**b**) Aptamer based multispot gold-capped NPs array (MG-NPA) chip containing a dielectric layer of a thin gold (Au) layer on silica (Si) NPs-absorbed glass slide (adapted with permission from [42]); (**c**) Measurements of *S. aureuse* on Au surface functionalized with grahene and aptamer with modification steps: (**A**) immobilization of mercaptobenzenediazonium tetrafluoroborate (MBDT) on grapheme; (**B**) Immobilization of graphene on Au; (**C**) immobilization of aptamer and (**D**) detachment of aptamers from graphene in the presence of *S. aureus* (adapted with permission from [38]).

GO was used in conjunction with AuNPs to fabricate an impedimetric aptasensor *S. aureus* on a glassy carbon electrode (GCE) [46]. The aptamer was immobilized via thiol chemistry on AuNPs (Figure 3a). Bacteria were quantified in the concentration range from 10 to 10^6^ CFU/mL with a detection limit of 10 CFU/mL (S/N = 3). Detection was also achieved by immobilizing different types of thiolated aptamers specific to *Lactobacillus acidophilus*, *Salmonella typhimurium* and *Pseudomonas aeruginosa* on a multispot gold-capped NPs array (MG-NPA) chip [51]. The chip was fabricated from a dielectric layer of a thin gold layer on silica NPs over a glass slide. Detection was achieved by measuring changes in the localized surface plasmon resonance (LSPR) upon the binding of bacteria (Figure 3b). In another work [47] an aptamer/graphene interdigitated gold piezoelectric sensor was fabricated using mercaptobenzenediazonium tetrafluoroborate (MBDT) attached to graphene on a gold surface through thiol chemistry. 

**Figure 4:**
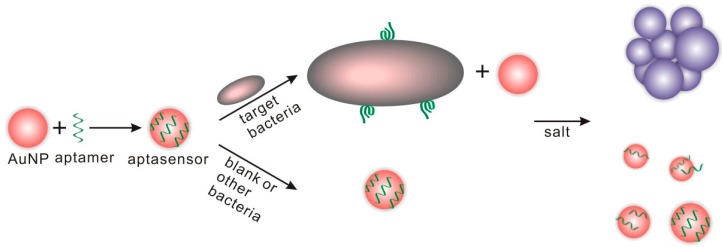
Colorimetric detection of bacteria using aptamers and AuNPs (adapted with permission from [41]).

Aptamers were immobilized on the surface of graphene through *π*–*π* stacking between aptamer bases and graphene. Upon the addition of *S. aureus*, the aptamer binds to its target (*S. aureus*) which causes detachment of the aptamer from the graphene surface causing a change in the oscillator frequency. Pathogen detection was completed within one hour. The sensor showed a linear relationship in concentrations ranging from 4.1 × 10 to 4.1 × 10^5^ CFU/mL *S. aureus* with a detection limit of 41 CFU/mL (Figure 3c). Similar concepts were explored using reduced GO and carboxyl-modified multi-walled carbon nanotubes (MWCNTs) electrochemically immobilized on the surface of a GCE and functionalized with an amino-modified aptamer specific for *Salmonella* [61]. When exposed to samples containing *Salmonella*, the anti-*Salmonella* aptamer on the electrode captures its target and the electron transfer is blocked, which results in a large increase in impedance. *Salmonella* was quantified in the range from 75 to 7.5 × 10^5^ CFU/mL with a detection limit of 25 CFU/mL.A popular strategy is to use AuNPs functionalized with aptamers and detect bacteria by colorimetric means by measuring aggregation/de-aggregation upon target binding [50]. Addition of target bacteria (*E. coli O157*:*H7* or *Salmonella typhimurium*) to AuNPs functionalized with their aptamers induce NP aggregation in presence of salt. Bacteria are quantified by a shift in color from red to blue (Figure 4). Using this strategy, aptamer-gold NPs sensors were designed for *E. coli O157*:*H7* and *Salmonella typhimurium*. The test was completed within 20 min or less in a concentration range close to 10^5^ CFU/mL [50]. Colorimetric visualization of bacteria holds promise as an attractive method to design instrument free, portable and simple to perform analysis that can be quantified by the naked eye or with simple color measurement software. Bacteria measurements have been reported with a variety of gold nanostructures including nanocrystals [25], nanostars [82], nanorods [83] and various aptamer-modified AuNPs [26,28,84]. A GCE modified with an immuno-double-layer of AuNPs and chitosan was used for *Bacillus cereus* detection*.* The results showed a high sensitivity of 10.0 CFU/mL [62]. Other materials such as lipopolysaccharides (LPS)-binding aptamer on the surface of nanoscale polydiacetylene (PDA) vesicle were also reported [49]. 

Fluorescence detection was achieved with CdTe QDs as fluorescent markers coupled with aptamers for selective binding and molecular recognition [19,45]. Specific recognition of *Vibrio parahaemolyticus* and *Salmonella typhimurium* from complex mixtures including shrimp samples was achieved using this aptamer-modified QDs and flow cytometer [42]. Simultaneous detection of these two bacteria was further demonstrated by using fluorescence resonance energy transfer (FRET) from green-emitting QDs and red-emitting QDs as donors, and amorphous carbon NPs as acceptors [19]. In the absence of target, the fluorescence of QDs is quenched by the carbon NPs. When a target is present, quenching is suppressed and this emitted light is related linearly to the concentration of bacteria. The concentration of the two pathogens measured by this method was from 50 to 10^6^ CFU/mL, with detection limits of 25 CFU/mL for *V. parahaemolyticus*, and 35 CFU/mL for *S. typhimurium*. Multiplexed analysis of *Staphylococcus aureus*, *Vibrio parahemolyticus*, and *Salmonella typhimurium* was demonstrated using rare earth upconversion NPs (UCNPs-NaYF4: Yb, Tm NaYF4: Yb, Ho, NaYF4: Yb, Er/Mn) as luminescence labels for aptamers. 

**Figure 5:**
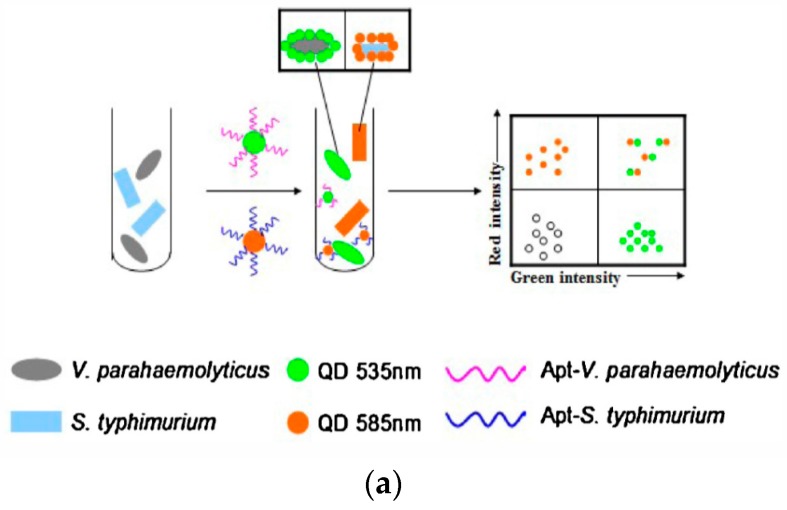

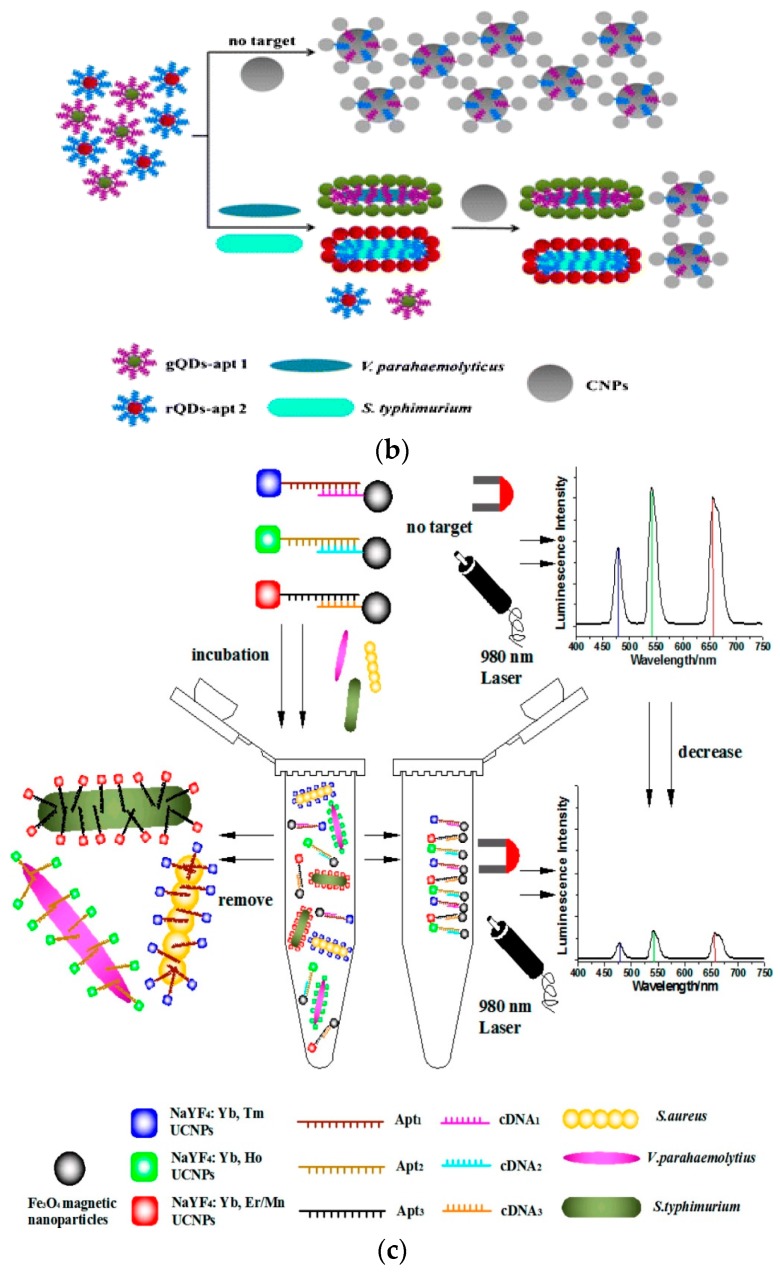
(**a**) Detection of *Vibrio parahaemolyticus* and *Salmonella typhimurium* using aptamer-functionalized QDs and flow cytometry (adapted with permission from [36]); (**b**) FRET based measurement b (adapted with permission from [31]); (**c**) shows multiplexed detection of three types of bacteria by three different aptamer-based upconversion rare earth NPs. The detection is based on the luminscence signals of free NPs after separation from bacteria mixture (adapted with permission from [39]. Copyright (2014) American Chemical Society).

For *S. aureus*, carboxylic acid-modified NaYF_4_: Yb, Tm UCNPs (UCNPs_Tm_) were conjugated with amino-modified Apt_1_ through carbodiimide chemistry, Fe_3_O_4_ magnetic nanoparticles (MNPs) were conjugated with cDNA_1_, and then Apt_1_-UCNPs_Tm_ was conjugated with cDNA_1-_MNPs. Before incubation with *S. aureus*, the collected luminescence signal was for UCNPs_Tm_-MNPs. After addition of *S. aureus*, Apt_1_ dissociated from UCNPs_Tm_ and attached to *S. aureus*. The emission peak was quenched as a result of the reduced concentration of UCNPs_Tm_-MNPs signal. The three types of UCNPs resulted in different colors which allowed multiplex analysis of pathogenic bacteria [48] as shown in Figure 5c. Other examples of fluorescence methodologies and the use of fluorescence labels for pathogen detection have been reviewed [85,86]. 

**Figure 6:**
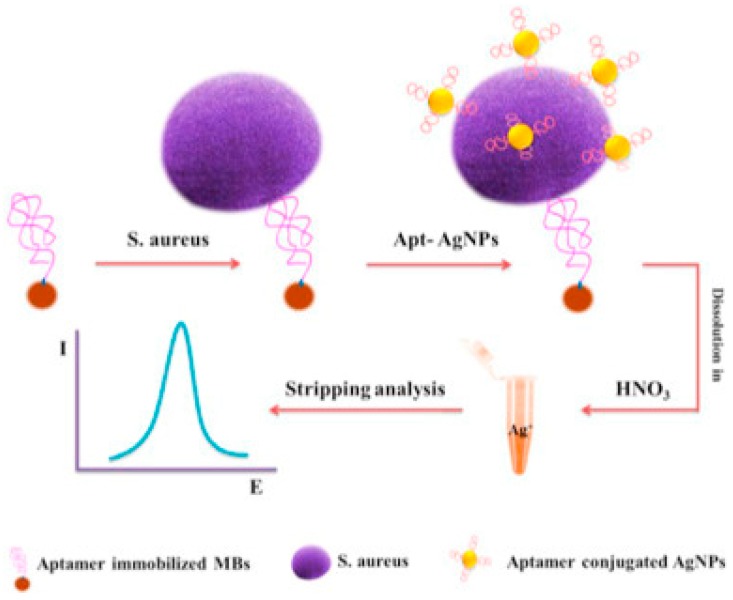
Example of electrochemical aptamer-based sensor with AgNPs labels and magnetic separation for detection of *S. aureus* (adapted with permission from [35]).

NP-based aptasensors have been connected with immunomagnetic platforms for separation and detection. A selective aptasensor with immunomagnetic separation and electrochemical detection was developed using a dual aptamer system with an aptamer for *S. aureus* attached to AgNPs and a primary aptamer attached to magnetic beads (MB) [44]. The capture probe consisted of a biotinylated primary anti-*S. aureus* aptamer attached to streptavidin-modified MB while AgNPs conjugated to a secondary aptamer were used for signal quantification. Bacteria in the sample will attach to aptamer-MB. The aptamer-AgNP will then bind to the MB carrying the bacteria, which is then separated by an external magnetic field. Finally, differential pulse stripping voltammetry was used to measure the bound AgNPs (Figure 6) A detection limit of 1.0 CFU/mL and a dynamic range from 10 to 1 × 10^6^ CFU/mL were reported for *S. aureus* in a sandwich format by measuring the electrochemical signal of AgNPs using anodic stripping voltammetry. Additionally, a florescent aptasensor for simultaneous detection of the pathogens *Vibrio parahaemolyticus* and *Salmonella typhimurium* was developed using carbon dots [19,87]. Improved stability was reported with an electrochemical aptamer sensor developed on a nanostructured gold microelectrode [88] fabricated by electrodeposition of dendritic-like gold structures.

### 3.2. Immuno-Based Nanosensor Strategies

**Figure 7:**
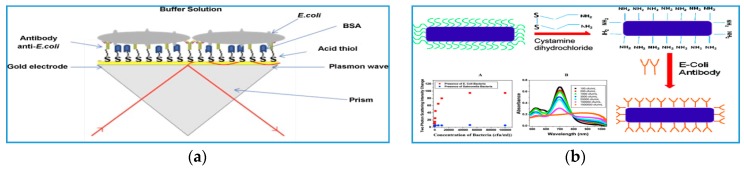
Examples of *E. coli* immunosensors using: (**a**) AuNPs with SPR quantification (adapted with permission from [14]) and (**b**) Au nanorods and two-photon Rayleigh scattering (TPRS) spectroscopy as a detection technique. Adapted with permission from [18]. Copyright (2009) American Chemical Society.

Conventional ELISA (enzyme-linked immunosorbent assay) assays utilize immunological reagents to detect bacteria [89]. Following similar principles, a variety of sensing platforms have been designed that incorporate antibodies (Ab) to improve portability, reduce analysis time and simplify detection [90]. Most nano-sensing platforms are based on AuNPs modified with Ab and detection is based on measurements of the surface plasmon resonance (SPR) or color change associated with aggregation/de-aggregation upon target binding. Figure 7 shows a SPR-based immunosensor for detection of *E. coli* K12 and *Lactobacillus fermentium* [23]. Antibodies specific for these bacteria were immobilized over a gold layer or AuNPs deposited atop the gold layer using 16-mercaptoundecanoic acid and carbodiimide coupling between the acid group on Au surface and the amine residue of the Ab. Measurements of the change in resonance angle and refractive index with different bacteria concentration provided a detection limit of 10^3^ CFU/mL when AuNPs were used, as compared to 10^4^ CFU/mL in the absence of NPs [23]. Singhet al. [27] have reported an immunosensor for detection of *E. Coli* using Au nanorods functionalized with *E. coli* Ab and two-photon Rayleigh scattering (TPRS) spectroscopy as a detection technique. In presence of *E. coli* O157:H7 bacterium*,* the modified nanorods bind to *E. coli* causing aggregation which resulted in an increase in the TPRS signal. The analysis took 15 min and the LOD was 50 CFU/mL. 

**Figure 8:**
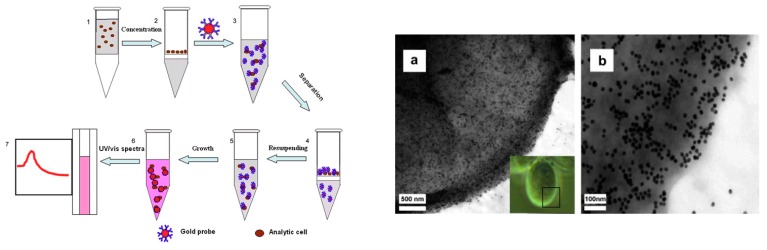
AuNPs-based immunosensor for Giardia lamblia cysts detection. (**Left**) The sample is concentrated through a centrifuge filter, and then incubated with Ab-AuNPs immunoprobes. The binding is quantified as a color change of the AuNPs detected by UV-spectroscopy. The (**Right**) image shows TEM images of immunoprobes on the surface of Giardia lamblia cysts at a scale of (**a**) 500 nm and (**b**) 100 nm. Giardia lamblia cysts morphology is shown in the inset (adapted with permission from [16]).

A colorimetric AuNPs-based immunosensor assay for *Giardia* *lamblia* *cysts* was developed using Ab-functionalized NP probes [25]. To perform the assay, bacteria were first concentrated on a centrifuge filter and then incubated with the immunoprobes (Figure 8). Binding was confirmed by TEM imaging. Unbound probes were removed by filtration. The color change of AuNPs due to binding was detected by UV-spectroscopy by measuring the red-shift UV-absorbance which showed an increased absorbance at 550 nm as bacteria concentration increases. A linear concentration range up to 10^4^ cells/mL was measured with a LOD of 1.088 × 10^3^ cells/mL. Other examples of AuNPs based detection are summarized in Table 2.

**Figure 9:**
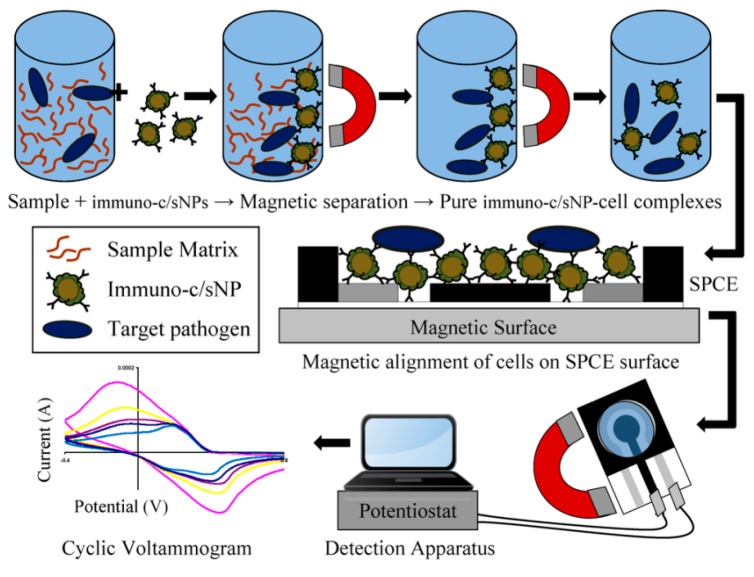
Schematic representation of the use of immuno-magnetic/polyaniline core/shell nanoparticle (c/sNP) with cyclic voltammerty for *Bacillus* and *E. coli* O157:H7 detection (adapted with permission from [17].

Setterington and Alocilja [26] designed an electrochemical immunosensor with magnetic separation for detection of *Bacillus* and *E. coli* O157:H7 using trifunctional NPs of immuno-magnetic/polyaniline core/shell (c/sNP). The NP system contains Abs as a specific bioreceptor for bacteria, a magnetic moiety to enhance separation and concentration and polyaniline as an electrical conductive material to enhance the conductivity for electrochemical measurements. The sensor was characterized by LODs of 40 and 6 CFU/mL for both bacteria types (Figure 9). Cyclic voltammetry and amperometry were used as detection techniques, showing a current decrease with increasing bacteria concentration.

Other works reported a low cost paper-based technology in which nitrocellulose paper was modified with immunological reagents against bacteria and AuNPs for detection. Li et al. [24] reported a multiplex paper-based immunosensor for detection of *Pseudomonas aeruginosa* and *Staphylococcus aureus* in which bacteria Abs were attached to AuNPs on nitrocellulose paper. The assay was developed as a portable strip reader and was able to detect 500–5000 CFU/mL. In other examples electrochemical immunosensors for *Salmonella* were designed using graphene quantum dots (GQDs) [91,92]. In other works, Deisingh and Thompson exploited the use of Raman spectroscopy on nano-engineered surfaces for bacterial detection in food and environmental analysis [93,94]. Other platforms use silica NPs as immobilization platforms for the detection of *Escherichia coli* [95]. Immobilization of the biosensing element on nanomaterials was shown to enhance the molecular recognition and increase the selectivity [68,96,97,98,99,100]. Three-dimensional modeling was used for nano-manipulations and predicting the selectivity towards different analyes [99,101,102]. The current development status demonstrates that immunosensors and aptasensors have great potential to improve performance of devices for pathogen detection and this approach can resolve a potentially large number of challenges in bioassays [103]. However, during the immobilization process, the lack of orientation of the antibodies or aptamers, which may result in random conjugation with the target of interest, are critical issues that still need to be addressed. Additional challenges are issues of specificity, some due to the presence of non-specific adsorption which require development of suitable materials and methods to improve selectivity, enable site-specific orientation of bio-receptors on surfaces and prevent non-specific adsorption. 

### 3.3. Phage-Based Recognition

**Figure 10:**
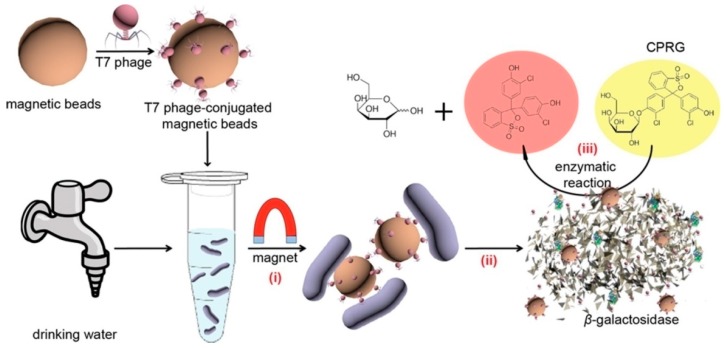
Schematic representation of the use of T7 bacteriophage-conjugated magnetic probe to detect *Escherichia coli* in drinking water (**i**) Introduction of T7 bacteriophage-conjugated magnetic probe to attack *E. coli* and separate it by the influence of magnet (**ii**) The explosion or lysis of *E. coli* and the release of T7 phages and β-gal; (**iii**) β-gal catalyzed CPRG hydrolysis to produce colorimetric signal (adapted with permission from [26]. Copyright (2015) American Chemical Society).

Bacteriophages or phages are viruses able to recognize and infect host bacteria producing a large number of virons and cause lysis to the host bacteria [104,105]. Phages are used to identify bacteria and differentiate between different types of bacteria strains [106]. They have a high specificity for their hosts, are able to differentiate between live and dead cells [42] and can be easily prepared at low cost [104,105]. These properties make phages good candidates as molecular recognition elements for designing biosensors for detecting bacteria. Table 3 summarizes the various types of phage based biosensors for bacteria and their transduction method reported in literature. Chen et al. [35] reported a multifunctional T7 bacteriophage-conjugated magnetic probe that was used to concentrate, separate and detect *Escherichia coli* (*E. coli*) from drinking water. The detection principle is shown in Figure 10. First, T7 bacteriophage was amino modified in order to attach to the carboxylic functionalized magnetic beads. Then, the T7 bacteriophage-conjugated magnetic probe was added to a sample containing *E. coli* and the *E. coli*-T7 bacteriophage-conjugated magnetic assembly was separated using a magnet. *E. coli* was lysed and β-galactosidase (β-gal) was released from bacterial cells. The signal was obtained by analyzing the catalytic process of β-gal to chlorophenol red-β-d-galactopyranoside (CRPG). The colorimetric signal was analyzed by UV spectrometry and a mobile camera. The method was able to detect *E. coli* at LOD of 1 × 10^4^ CFU/mL within 2.5 h. The specificity of the phage based magnetic probes toward *E. coli* was demonstrated against *Salmonella enterica* (*S. enterica*), *Staphylococcus aureus* (*S. aureus*), and *Pseudomonas aeruginosa* (*P. aeruginosa*). A concentration of 10 CFU/mL in drinking water was detected after 6 h pre-enrichment. Several other studies reported similar designs with optical transduction methods [35,36,43]. Quantitative details on these sensors are summarized in Table 3.

Olsen et al. 2006 [40] prepared a biosensor to detect *Salmonella typhimurium* by using physically adsorbed bacteriophage on a piezoelectric transducer. Upon bacteria binding, a decrease in resonance frequency occurs, allowing quantitative measurement of the bacteria host by the immobilized bacteriophage. Guntupalli et al. [38] studied the detection and differentiation between methicillin resistant (MRSA) and sensitive (MSSA) *Staphylococcal* species using QCM with dissipation (QCM-D). Detection was achieved using immobilized lytic phages on the QCM sensor. 

**Figure 11:**
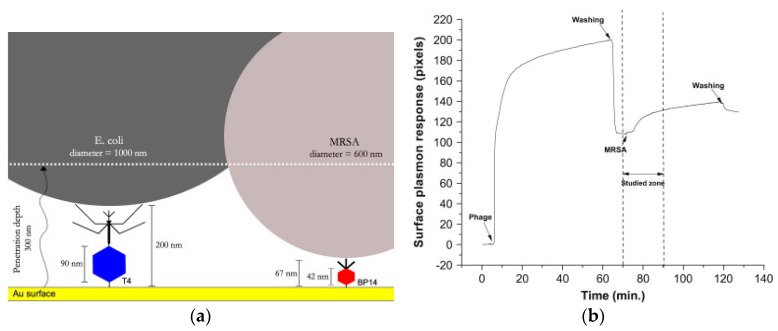
(**a**) Concept of bacteriophage-based sensor for *E. coli* and MRSA using covalently attached T4 and BP14 bacteriophages and (**b**) The response of SPR upon attachment of phages, and then with *E. coli*. (adapted with permission from [42]).

The binding of bacteria to phage resulted in reduced frequency and increased dissipation energy. MRSA and MSSA strains were differentiated by exposure to penicillin-binding protein Ab after binding to phages. MRSA interacts with Abs due to their specificity, while MSSA didn’t. Another bacteriophage sensor was developed for detection of *E. coli* and MRSA using SPR detection [42]. T4 bacteriophage was covalently attached to gold surface for *E. coli* and a specific bacteriophage B14 was used for MRSA detection (Figure 11). BSA was added to prevent non-specific adsorption. Contact of bacteria with the phage initiated bacteria lysis within 20 min producing a concentration-dependent change in the SPR signal after 10 min. 

**Figure 12:**
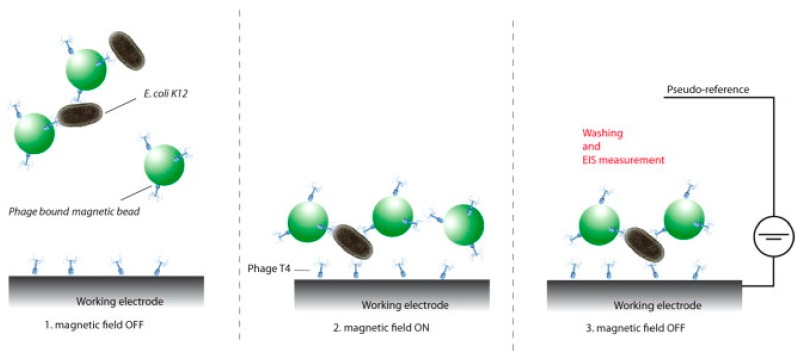
Illustration of the screen-printed carbon electrode and its use for the EIS detection of magnetically separated *E. coli* K12 using immobilized bacteriophages (adapted with permission from [32]).

In other bacteriophages-sensing designs, lysis products were quantified by electrochemical techniques with sensors based on amperometric [39] and impedimetric detection [41]. For example, the presence of β-d-Galactosidase enzyme in the lysis products was used to quantify *E. coli*. The enzymatic activity was measured amperometrically using p-aminophenyl-β-d-galactopyranoside (β-PAPG) as substrate and determining the product of the reaction, p-aminophenol through oxidation at a carbon electrode [39]. Direct impedance measurements of bacteria were accomplished by using phages as recognition probe without directly quantifying lysis components [38,40,41,42]. T4 phage was directly immobilized onto screen-printed carbon electrode microarrays using magnetic beads, to act as a specific probe [41] as shown in Figure 12. 

The phage-modified beads were then mixed with the bacteria sample for 10 min, and the mixture was deposited onto to the phage-modified screen-printed electrode. A magnet was then placed under the electrode to attract the magnetic beads, along with the captured bacteria. The amount of bacteria captured by the phage was measured by impedance spectroscopy.

### 3.4. Molecularly Imprinted Polymers (MIP)

Molecularly imprinted polymers defined as artificial recognition elements are of growing interest for applications in several life science sectors involving the separation and detection of specific molecules [107,108]. These polymers have attractive properties such as high recognition capability, mechanical and chemical stability, easy preparation and low cost which make them superior over natural recognition reagents [107,109]. Sensing of pathogens is also possible using molecular imprinting that allows creation of specific recognition sites by polymerization of monomers in presence of a template molecule [110]. Removal of the template creates a shape memory cavity with binding properties that can serve as recognition sites for molecules with identical geometry to that of the imprint molecule [111,112]. The main advantages of this approach are the stability and low cost [111], and the ability of several analytes to be bounded to MIPs ranging from small to large molecules [58,59,60,113,114]. Target binding can be monitored using electrochemical, QCM or SPR methods. 

**Figure 13:**
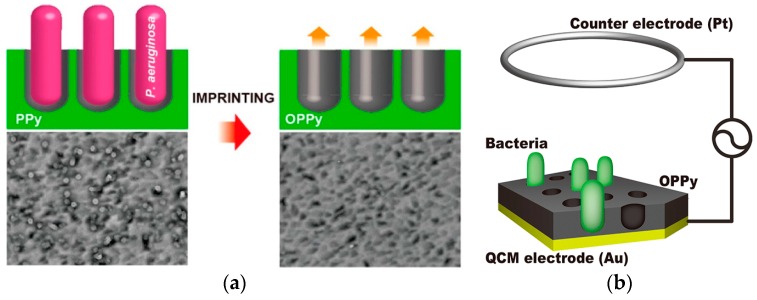
(**a**) Illustration depicting imprinting *Pseudomonas aeruginosa* bacteria on a polypyrrole (PPy) film and (**b**) electrode configuration for label free detection with the PPy film (adapted with permission from [50]. Copyright (2013) American Chemical Society).

A QCM-based MIP bacteria platform was reported using electrochemically polymerized polypyrrole (PPy) deposited as a thin film on gold-evaporated quartz crystal [59]. The bacteria was removed by applying lysozyme and 10% Triton X to disrupt the binding between the bacteria’s polysaccharide surface and the polymer and then overoxidized leaving a shape memory cavity for bacteria. Living bacteria were trapped vertically in the cavity (Figure 13) and quantified using QCM by measuring the decrease of oscillating frequency upon exposure to bacteria. The method showed high selectivity to *Pseudomonas aeruginosa*, *Acinetobacter calcoaceticus, E. coli*, and *Serratia marcescens* and was demonstrated in apple juice as a real sample. A detection limit of 10^3^ CFU/mL and a linearity range from 10^3^ to 10^9^ CFU/mL was obtained within 3 min, without any pretreatment.

**Figure 14:**
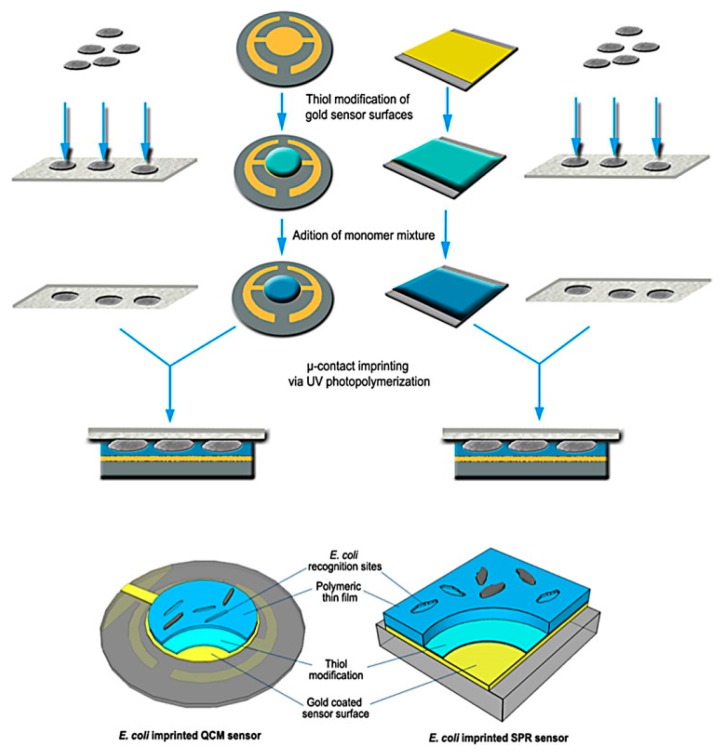
Schematic representation of micro-contact imprinted SPR and QCM sensor surfaces (adapted with permission from [60]).

A MIP-based QCM and SPR detection [60] of *E. coli* was developed on a modified gold surface. QCM and SPR gold surfaces were modified with allyl mercaptan and *N*-methacryloyl-*l*-histidine methylester monomers that have some similarities with natural antibodies. Micro-contact imprinting of *E. coli* was achieved by UV-photo polymerization as shown in Figure 14. Bacteria were removed using lysozyme. The sensors showed short response times of 113 s for SPR and 56 s for QCM were used with apple juice as real sample. However, the LOD of both methods were relatively high of 1.54 × 10^6^ CFU/mL, 3.72 × 10^5^ CFU/mL with SPR and QCM respectively.

Jiang et al. [58] demonstrated detection of some types of Gram-negative bacteria by measuring their quorum signaling small organic molecules N-acyl-homoserine-lactones (AHLs) and templated magnetic Fe_3_O_4_ to facilitate separation. The MIP sensor was fabricated using 2,5-dimethyl-4-hydroxy-3(2*H*)-furanone (DMHF) selected as a template due to its chemical and size similarity to AHL. Measurements were run in a solution of Fe(CN)_6_^3−/4−^ using cyclic voltammetry (CV) and differential pulse voltammetry (DPV). The method enabled detection of AHL with a detection limit of 8 × 10^−10^ mol/L and a linear detection range from 2.5 × 10^−9^ mol/L to 1.0 × 10^−7^ mol/L. The design was successful in detecting *Aeromonas hydrophila* and *Pseudomonas aeruginosa.* Differentiation between strains with and without AHL was also demonstrated. A summary of MIP-based sensors for pathogen detection is provided in Table 4. 

### 3.5. Antimicrobial Peptides

Naturally occurring antimicrobial peptides (AMPs) are part of the host’s innate immune system acting as a defensive mechanism against invasive species [115]. The antimicrobial activity is thought to originate from binding to the bacteria surface and disruption of the cell membrane [116]. Although most applications of AMPs are in the clinical field [117,118] a few studies have explored the recognition properties of AMPS for bacteria detection [119,120,121,122]. Recent work has demonstrated the capability of both natural and synthetic antimicrobial peptides (AMPs) to act as biorecognition elements for the detection and differentiation of bacteria. In most designs, impedance spectroscopy has been used to monitor binding of bacteria to electrode surfaces. AMPs provide high stability and good activity even under harsh environments [123,124]. Their main disadvantage is the lack of selectivity. As alternative to natural peptides, it is possible to rationally design synthetic peptides with improved binding characteristics. The advantages of the synthetic AMPs are the possibility to rationally design their structure, binding and recognition properties as well as their low cost production and high stability [123,124]. 

We have recently used synthetic engineered supramolecular AMPs to design an impedimetric biosensor for detection of bacterial pathogens [56]. The biosensor was developed on a AuNPs-functionalized electrode that was modified with synthetic AMPs through site specifically engineered amino acids which enabled oriented attachment of the AMPs. The peptides were synthesized from a beta-sheet-forming peptide, K_2_(QL)_6_K_2_ that showed antimicrobial activity against *E. coli*, *Pseudomonas aeruginosa* (*P. aeruginosa*), *Staphylococcus aureus* (*S. aureus*) and *Staphylococcus epidermidis* (*S. epidermidis*) [125,126].

**Figure 15:**
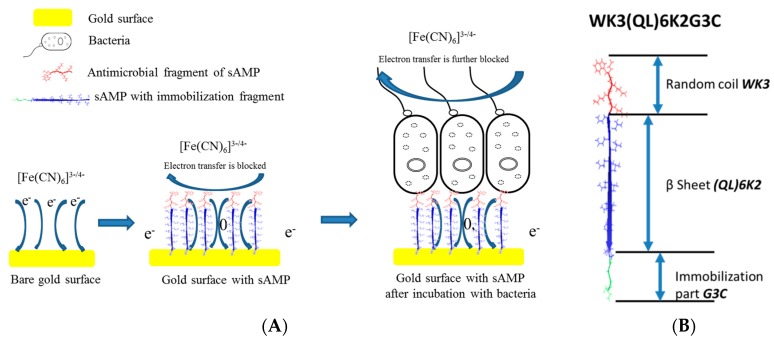
(**A**) *Impedimetric* bacteria sensing platform using synthetic cysteine-modified AMP. (**B**) Sequence and orientation of the active peptide (WK_3_(QL)_6_K_2_G_3_C) on AuNPs functionalized electrode (adapted with permission from [56]).

To enable controlled binding, the peptides were modified with an external cysteine residue that allowed one step site-specific orientation to a gold surface through the affinity of cysteine for gold. The peptide structure and biosensor design are illustrated in Figure 15. EIS was used as transduction method to quantify the binding of bacteria and enable rapid and label free detection in a single step. This strategy could be used in the future to prepare sensor chips for high-throughput screening of bacteria [127]. The method can also be used to modify surfaces to impart antimicrobial activity for detection and prevention of biofilm formation. 

**Figure 16:**
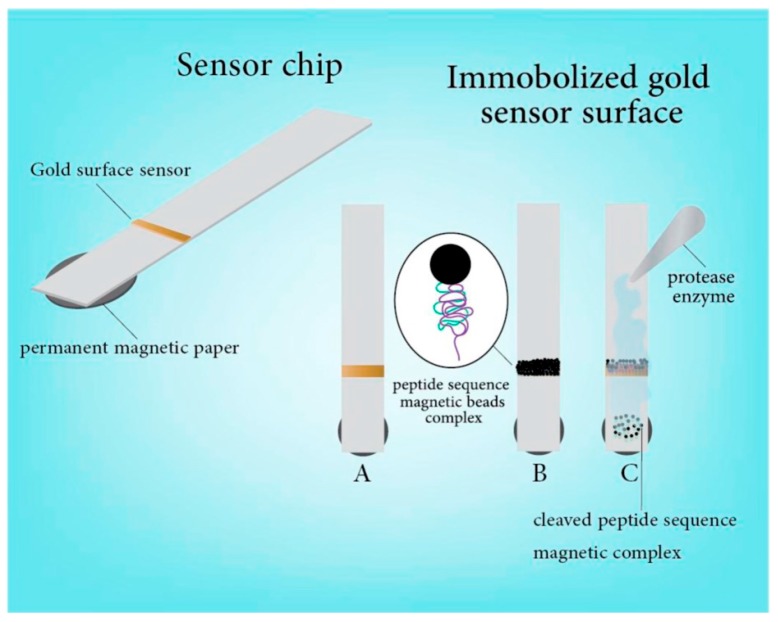
Listeria biosensor using modified magnetic NP. (**A**) gold sputtered on paper (yellow) over a plastic strip with a magnet underneath to remove unbounded magnetic NPs after immobilization; (**B**) magnetic NPs with immobilized peptide sequence placed over the gold surface to mask the color; (**C**) Adding protease enzyme of *L. monocytogenesis* will cleave the peptide from the NPs resulting in dissociation of the magnetic beads complex, exposing the gold surface (adapted with permission from [128]).

Other mechanisms involve the use of proteases attached on magnetic NP surfaces. Alhogail et al. [128] reported a colorimetric assay for *Listeria* using a specific magnetic NPs-protease-gold sensing probe (Figure 16). Magnetic NPs were conjugated with a *L. monocytogenes* protease specific substrate which selectively cleaves the *L*. *monocytogenes* proteases. The substrate was linked to carboxylated magnetic NPs using carbadiimide chemistry, which were then deposited onto a gold sensor surface on paper forming black magnetic nanobeads. Detection was based on color change from black to golden upon the cleavage of the specific peptide sequence by *Listeria protease*. A LOD of 2.17 × 10^2^ CFU/mL was reported for *Listeria* with high specificity against four different foodborne bacteria (*E. coli*, *Salmonella*, *Shigella flexnerii* and *Staphylococcus aureus*). The sensor showed functionality in artificially spiked milk and ground meat. 

Other paper based sensors for bacteria were reported based on detection of enzyme activity. Jokerst et al. [129] and Adkins et al. [130] detected bacteria by measuring the change in color of the substrate due to enzyme evolution from the bacteria. Enzymes such as β-gal and β-glucuronidase (β-glucur) are both produced by *E. coli*, while β-glucosidase is produced by *Enterococcus* spp. Thus the enzymatic catalysis of *p*-nitrophenyl-β-d-glucuronide (PNP-glucer) into phenolic compound *p*-nitrophenol (PNP) by β-glucur is expected and can be measured as color change from colorless to yellow at pH > 7.18 [130]. 

### 3.6. Multifunctional Platforms for Inactivation and Detection of Pathogens

The development of multifunctional platforms for packaging applications is an area of growing interest. Most nanocomposites used for packaging are based on AgNPs [64,131,132,133] but other materials like GO [134], polyelectrolyte multilayers [135], antimicrobial polymer nanocomposites [136] and natural antimicrobial agents [69] have also been reported. Platforms carrying antimicrobial activity could be interfaced in the future with biomolecular recognition and be used to detect and control foodborne pathogens. 

**Figure 17:**
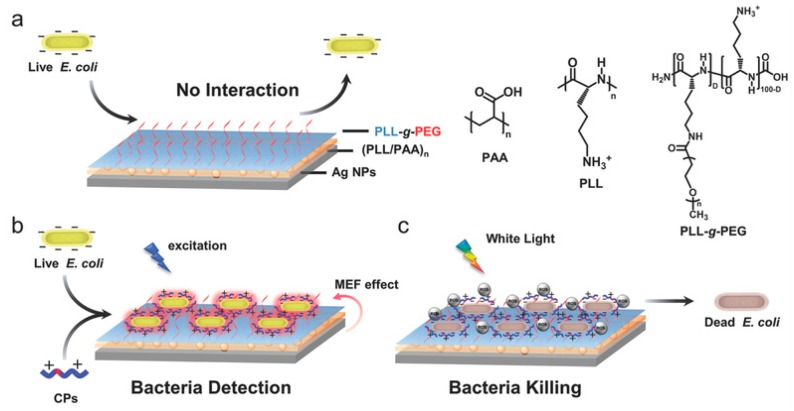
(**a**) Example of multifunctional bioassay for detection and disinfection using adsorbed PLL-g-PEG brushes for preventing adsorption of proteins and bacteria (**b**) Operational principle for capture and detection of *E. coli* by fluorescently measuring the response of CP coupled with the plasmon-enhance fluorescence from the Ag nanostructure (**c**) Disinfection is achieved from reactive oxygen species produced by CP under white light irradiation (adapted with permission from [18]).

Recent developments in pathogen detection are focused towards fabrication of integrated platforms that can perform multiple functions for simultaneous capture, detection and inactivation. Wang et al. [137] described a SERS multifunctional chip made of silicon wafer containing AgNPs modified with 4-mercaptophenylboronic acid (4-MPBA). The platform showed binding and detection capabilities for *E. coli* and *S. aureus* at a concentration range of 500–2000 CFU/mL (LOD was 200 CFU/mL) while also inactivating the pathogens on contact in human blood. Bacteria inactivation was enabled by the dissolved Ag^+^ ions released from the immobilized AgNPs. Multifunctional capabilities for capture, detection and inactivation were also reported with conjugated polyelectrolytes (CPs)-Ag nanostructures [18]. In this system, (Figure 17) detection and inactivation was achieved through the fluorescence and light-harvesting properties of CPs originating from their conducting polymer backbone which provide optical properties and the ability to generate reactive oxygen species (ROS), enhanced by the use of Ag as substrate. 

Other types of multifunctional platforms that integrate separation and concertation of the sample typically based on magnetic particles functionalized with Ab or aptamers are now commonly used to capture and separate targeted analytes in the presence of an external magnetic field. For example, aptamer-functionalized Fe_3_O_4_ magnetic NPs were used to separate *Staphylococcus aureus*, *Vibrio parahemolyticus*, and *Salmonella typhimurium* from solution [48]. In another case, FeCo NPs were used to separate phage bounded bacteria from the unbound bacteria [34]. A screen printed carbon electrode modified with immuno-magnetic/polyaniline core/shell NPs was also developed for separation and detection of *Bacillus cereus* and *E. coli* with LODs of 40 CFU/mL and 6 CFU/mL, respectively using immunomagnetic separation and electrochemical detection [26].

Simultaneous detection, elimination, and inactivation of pathogenic bacteria was illustrated using vancomycin-functionalised AgNPs/3D-ZnO nanorod arrays [138]. In the system, three-dimensional ZnO nanorod arrays were used as detection platform, AgNPs were used as antibacterial agent, while vancomycin was used to selectively recognize pathogenic bacteria. Moreover, a hybrid of multifunctional system consisting of graphene oxide/AgNPs (GO-Ag NPs) was synthesized and applied for monitoring and disinfecting of gram-negative *Escherichia coli* as well as the gram-positive *Staphylococcus aureus* [139]. In another work, a multifunctional nanosystem based on synthesized core–shell fluorescent magnetic NPs (FMNPs) conjugated with gentamicin were able to capture and disrupt the cell wall of *E. coli* (1 × 107 CFU mL^−1^ from 10 mL of solution) within 20 min [140]. 

## 4. Conclusions and Future Perspectives 

Biosensors for pathogens detection are widely used. Nanomaterials can provide optical, catalytic, magnetic and antimicrobial properties for sensing applications. Therefore, the integration of nanotechnology in sensing platforms has provided significant enhancements in detection capabilities and functionality of these devices. On the other hand, multifunctional nanosystems have the potential to act simultaneously as a method for rapid microbial capture, detection, and decontamination. Thus, future developments are also expected in the development of smart labels to indicate food spoilage or presence of harmful toxins. Hence, several types of NPs and nanocomposites have been used in the packaging industry to inhibit bacterial growth and increase the shelf-life of foods. Systems with integrated detection, capture and inactivation capabilities could be developed in the future to design multifunctional platforms for food safety applications. Consequently, the introduction of nanosensors to food packaging to indicate contamination, detect microorganisms, toxicants, moisture or gases from food spoiling is expected to grow. An area of future development is to design food packaging equipped with smart and connected indicators and nanosensors that can be used as tracking devices for product identification, authenticity, traceability and anti-counterfeiting [141]. To enable rapid implementation of this technology in consumer products, this area would benefit from fundamental advances in the development of low cost and flexible nano-sensors suitable for roll-to-roll manufacturing for large scale production. The use of inexpensive materials such as paper or plastic and integration of all sensing reagents (including standards) into a portable compact unit is also desirable for future deployment and rapid implementation of these devices. Method validation, comparability, stability and inter-laboratory studies to evaluate performance are also needed to ensure robustness and accuracy of these devices for real world applications. Eventually, application for the detection of pathogenic organisms in complex matrices needs to be demonstated in real samples to move this technology into the market place. 

## Figures and Tables

**Table 1:** Aptamer-based biosensors for bacteria detection.

Ref.	Target Bacteria	NPs Used in the Sensor	NPs Function	LOD	Real Sample	Time	Detection Method	Range
[53]	*Salmonella Typhibacteria*	SWCNT	Conductive support for aptamer where change in conformation occurs in presence of target bacteria	1 CFU/mL	-	Few seconds	Potentiometric	0.2–10^3^ CFU/mL
[52]	*E. coli CECT 675* as a nonpathogenic surrogate for pathogenic *E. coli O157*:*H7*	SWCNT	Conductive support for aptamer where change in conformation occurs in presence of target bacteria	LOD 12 CFU in 2 mL of milk and 26 CFU/mL in apple juice	Milk and apple juice	Couple of minutes	Potentiometric	linear response of up to 10^4^ CFU/mL
[50]	*E. coli* O157:H7 and *Salmonella typhimurium*	AuNPs	Color change due to target induced aggregation	10^5^ CFU/mL		20 min or less	Optical/Colorimetric UV-Vis	
[45]	*Vibrio parahaemolyticus* and *Salmonella typhimurium*	CDs	Fluorescent label	5 × 10^3^ CFU/mL	Shrimp		Optical/Fluorescence	3.8 × 10^4^–3.8 × 10^7^ CFU/mL
[54]	*Lactobacillus acidophilus, Staphylococcus aureus* and *Salmonella enterica*	Gaphene oxide (GO) nanomaterial	Fluorescent signal adsorbent	11.0 CFU/mL for *Lactobacillus acidophilus* 61.0 CFU/mL for *S. enterica* and 800.0 CFU/mL and *S. aureus*		10 min	Optical/Fluorescence	9.4–150.0 CFU/mL for *Lactobacillus acidophilus* 42.2–675.0 CFU/mL for *S. enterica* and 10^4^–10^6^ CFU/mL for *S. aureus*
[46]	*Staphylococcus aureus*	AuNPs-reduced graphene oxide nanocomposite	Signal-amplification and support for aptamer	10 CFU/mL	water and fish	60 min	Electrochemical/impedance	10–10^6^ CFU/mL
[49]	*E. coli* O157:H7	nanoscale polydiacetylene polymer (PDA )	Generates color change	10^4^ CFU/mL	Clinical fecal specimens	2 h	Optical/colorimetric UV-Vis	10^4^–10^8^ CFU/mL
[51]	*Lactobacillus acidophilus* *Salmonella typhimurium* *Pseudomonas aeruginosa*	Au layer	The combination of gold and silicon NPs (MG-NP) forms a dilectric layer; attachment of biomolecule changes the peak extinction intensity	30 CFU per assay	-	-	Optical/localized surface plasmon resonance LSPR	10^9^–10^4^ CFU/mL
[48]	*Staphylococcus aureus, Vibrio parahemolyticus, and Salmonella typhimurium*	1-Rare earth upconversion nanoparticles (UCNPs) (NaYF4: Yb, Tm NaYF4: Yb, Ho NaYF4: Yb, Er/Mn), 2-magnetic nanoparticles Fe_2_O_3_	1-luminescence labels for aptamers 2-separation and concentration	25, 10, and 15 CFU/mL for *S. aureus*, *V. parahemolyticus*, and *S. typhimurium,* respectively	Milk and shrimp	-	Optical/luminescence	50–10^6^ CFU/mL
[19]	*Vibrio parahaemolyticus* and *Salmonella typhimurium*	1-QDs 2-novel amorphous carbon nanoparticles (CNPs)	1-Fluorescence emitter 2-Fluoresence acceptor	25 CFU/mL for *V. parahaemolyticus*, and 35CFU/mL for *S. typhimurium*	Chicken and shrimps	-	Optical/dual fluorescence resonance energy transfer (FRET)	50–10^6^ CFU/mL
[47]	*Staphylococcus aureus*	graphene to interdigital gold electrodes connected to a series electrode piezoelectric quartz crystal	-	41 CFU/mL	Milk	60 min	Mechanical/series electrode piezoelectric quartz crystal SPQC	4.1 × 10^1^–4.1 × 10^5^ CFU/mL
[44]	*Staphylococcus aureus* (*S. aureus*)	AgNPs	Origin of electrochemical signal	1.0 CFU/mL	Real water	-	Electrochemical/stripping voltammetry	10–1 × 10^6^ CFU/mL
[28]	*Salmonella enterica serovar Typhimurium*	antibodies -horseradish peroxidase-gold nanoparticles	Amplification of color	1 × 10^3^ CFU/mL	milk	<3 h	Optical	1 × 10^3^–1 × 10^8^ CFU/mL
[61]	*Salmonella*	multi-walled carbon nanotubes (MWCNTs)	Signal-amplification and a support material for the bioreceptor (aptamer)	25 CFU/mL	chicken	60 min	Amperometric: Cyclic voltammetry and impedimetric	75–7.5 × 10^5^ CFU⋅mL^−^^1^

**Table 2:** Immuno-based biosensors for bacteria detection.

Ref.	Target Bacteria	NPs	NPs Function	LOD	Real Sample	Time	Detection Method	Range
[23]	*E. coli* K12 (gram negative) and *Lactobacillus fermentium* (gram positive)	AuNPs	amplifying the SPR signal	10^4^ CFU/mL and 10^3^ CFU/mL in presence of Au NPs	-	1 min	SPR	10^5^–10^7^ CFU/mL
[24]	*Pseudomonas aeruginosa* and *Staphylococcus aureus*	AuNPs	Signaling- origin of color	-	Sputum	5 min	Visually and Optical Density at 600 nm (OD_600_)	500–5000 CFU/mL
[25]	*Giardia lamblia* cysts	AuNPs	Signaling- origin of color	1.088 × 10^3^ cells mL^−1^	-	-	UV-Vis	10^3^–10^4^ cells/mL
[26]	*Bacillus* and *E. coli* O157:H7	magnetic/polyaniline core/shell nanoparticle (c/sNP)	Separation and electrical conductive based material	40 CFU/mL and 6 CFU/mL	-	~1 h	Amperometric: Cyclic voltammetry	10^0^–10^2^ CFU/mL
[27]	*E. coli O157:H7*	Au nanorods	Signaling- origin of color	50 CFU/mL	-	15 min	two-photon Rayleigh scattering (TPRS)	50–2100 CFU/mL
[62]	*Bacillus cereus*	AuNPs	Increase sensitivity and stability	10.0 CFU/mL	Milk	-	Amperometric: Cyclic voltammetry	5.0 × 10^1^–5.0 × 10^4^ CFU/mL

**Table 3:** Phage-based biosensors for bacteria detection.

Ref.	Phage	Target Bacteria	LOD	Sample	Time	Detection Method	Range
[35]	T7	*E. coli*	10 CFU/mL	Drinking water	2.5 h	Optical/colorimetric	-
[36]	M13KE phage	*E. coli* K12	5 CFU/L	Water	overnight	Colorimetric-culture based assay	-
			50 CFU/L water (or 5 CFU/mL orange juice and skim milk)	Water, orange juice and skim milk	<4 h	Colorimetric-solution based assay	-
[34]	*T7*	*E. coli* K12	-	-	-	Bacteria culture	10^2^–10^7^ CFU/mL
[43]	Engineered HK620	*E. coli* TD2158 and *Salmonella*	10 bacteria/mL	Sea water	1 h	Optical/Fluorescence	
[37]	Engineered HK620 and HK97	*E. coli*	10^4^ bacteria/mL	-	1.5 h	luminescence	-
[39]	virulent phage-typing (λ vir)	*E. coli* (*K-12*, *MG1655*)	1 CFU/100 mL	-	6–8 h	Electrochemical/amperometric	10^2^–10^5^ with extended incubation time and 10^5^–10^9^ without time extension
[41]	T4	*E. coli* K*12*	10^3^ CFU/mL	*Milk*	-	Electrochemical/impedimetric	10^3^–10^8^ CFU/mL
[40]	Filamentous phage (clone E2—displaying foreign peptide VTPPTQHQ	*Salmonella typhimurium*	10^2^ cells/mL	-	<180 s	Mechanical/QCM	10^1^–10^7^ cells/mL
[38]	*S. aureus* bacteriophage	*Staphylococcal* and methicillin resistant (MRSA) and sensitive (MSSA) *S. aureus* species	10^4^ CFU/mL surface plasmon resonance	-	16 min	Mechanical/QCM	-
[42]	T4 and BP14 phage was used to detect MRSA	*E. coli* O157:H7 and methicillin-resistant *Staphylococcus aureus* (MRSA)	10^3^ CFU/mL		20 min	Optical/SPR	-

**Table 4:** MIP-based biosensors for bacteria detection.

Ref.	MIP	Target Bacteria	NPs Used in the Sensor	NPs Function	LOD	Real Sample	Time	Detection Method	Range
[59]	Polypyrrole (PPy)	*Pseudomonas aeruginosa*	-	-	10^3^ CFU/mL	Apple juice	3 min	Mechanical/QCM	10^3^ to 10^9^ CFU/mL
[60]	-	*E. coli*	-	-	1.54 × 10^6^ CFU/mL, 3.72 × 10^5^ CFU/mL with SPR and QCM	Apple juice	113 s for SPR 56 s for QCM respectively, while respectively.	1-Optical/SPR 2-Mechanical/QCM	5.13 × 10^6^ CFU/mL, 1.24 × 10^6^ CFU/mL with SPR and QCM
[58]	2,5-dimethyl-4-hydroxy-3(2H)-furanone (DMHF)	*Aeromonas hydrophila* and *Pseudomonas aeruginosa*	Magnetic Fe_3_O_4_@SiO_2_–NH_2_ (MNPs)	Faciliate separation	AHL LOD 8 × 10^−10^ mol L^−1^	Bacteria supernatant spiked samples	-	Electrochemical/ Differential Pulse Voltammetry (DPV)	2.5 × 10^−9^–1.0 × 10^−7^ mol/L
